# Structural reversal of disc cupping measured in Bruch’s membrane opening-based OCT morphometry after PRESERFLO microshunt implantation for open-angle glaucoma

**DOI:** 10.1186/s12886-024-03838-3

**Published:** 2025-01-17

**Authors:** Jan Niklas Lüke, Constantin Popp, Caroline Gietzelt, Florian Steinberg, Vincent Lüke, Alexandra Lappa, Thomas Dietlein, Philip Enders

**Affiliations:** 1 Department of Ophthalmology, Medical Faculty, University Hospital of Cologne, Kerpener Strasse 62, 50937 Cologne, Germany; 2 Department of Ophthalmology Medical Faculty, University Hospital of Cologne, Kerpener Str. 62, 50937 Cologne, Germany

**Keywords:** Coherence tomography, Intraocular pressure, Glaucoma, Structural reversal, Rebound tonometry

## Abstract

**Background/ Aims:**

To analyze the longitudinal change in Bruch’s membrane opening minimal rim width (BMO-MRW) and peripapillary retinal nerve fiber layer (pRNFL) thickness using optical coherence tomography (OCT) after implantation of a PRESERFLO® microshunt for surgical glaucoma management in adult glaucoma patients.

**Methods:**

Retrospective data analysis of 59 eyes of 59 participants undergoing implantation of a PRESERFLO microshunt between 2019 and 2022 at a tertiary center for glaucoma management. Surgical management included primary temporary occlusion of the glaucoma shunt to prevent early hypotony. Pre- and post-operative OCT examinations of the optic nerve head (ONH) and intraocular pressure (IOP) were assessed. Longitudinal change in morphometric spectral domain OCT parameters of the ONH was correlated to change in IOP.

**Results:**

BMO-MRW increased significantly between baseline (BL) and follow-up (FU) within the first three months after surgery (BL = 171.15 ± 66.80 μm; FU = 180.78 ± 70.394 μm; *p* = 0.034). For the same postoperative period, the mean preoperative IOP of 24.97 ± 7.22mmHg was lowered after surgery to 13.70 ± 5.09 mmHg. Eighteen months after surgery, there was no significant change in BMO-MRW compared to baseline (BL = 169.83 ± 52.69 μm; FU = 164.98 ± 55.85 μm; *p* = 0.271), while mean IOP was 13.08 ± 4.48 mmHg. A decrease in IOP correlated significantly with a change in BMO-MRW (*r* = 0.453, *p* < 0.05) three months after surgery. Peripapillary RNFL thickness was unchanged in follow-up after three months (*p* > 0.16) and significantly decreased in later follow-up (*p* = 0.009).

**Conclusion:**

PRESERFLO® microshunt implantation with primary temporary occlusion leads to a significant transient increase in BMO-MRW. This phenomenon is also known as structural reversal of disc cupping (SRDC). The effect seems to be less pronounced and of shorter duration when compared to previous data after trabeculectomy with comparable pre- and postoperative IOP levels.

## Introduction

The implementation of spectral-domain optic coherence tomography (SD-OCT) in glaucoma diagnostics represents a paradigm change in the evaluation of the progression of glaucoma [1]. Both OCT parameters peripapillary retinal nerve fiber layer (RNFL) thickness and Bruch membrane opening minimum rim width (BMO-MRW) to assess neuroretinal rim tissue appear to incorporate superior diagnostic power compared to parameters obtained by confocal laser scanning tomography (CSLT) [2]. When comparing the two SD-OCT parameters, RNFL thickness showed slight advantages in the correlation between functional visual field loss and morphologically measured loss of neuroretinal tissue [3]. RNFL thickness and Bruch-membrane-opening-based (BMO) parameters have similar high intraday reproducibility and do not appear to correlate with normal diurnal variations in intraocular pressure [4].

However, sudden changes in intraocular pressure (IOP) have been shown to significantly influence the morphometry of the optic nerve head (ONH): Structural reversal of optic disc cupping (SRDC) describes an increase in thickness of ONH’s neuroretinal rim after glaucoma surgery. The phenomenon, initially discovered as funduscopic observation, could be objectified both by CSLT and SD-OCT later [5, 6]. In some patients, the postoperative increase of BMO-MRW thickness was seen for more than twelve months after surgery [7].

Filtrating glaucoma surgery represents the most important approach in the surgical treatment of moderate to advanced open-angle glaucoma when the IOP can not be controlled sufficiently by topical IOP lowering medication. For many glaucoma surgeons, trabeculectomy with mitomycin remains the gold standard in filtrating surgery, mainly because of the higher rate of success compared to other approaches [8, 9].

Alternative surgical techniques aim to reduce side effects and risks of trabeculectomy with mitomycin, mainly early hypotony. Minimally invasive shunts aim to bypass the anterior chamber fluid into the subconjunctival space. The PRESERFLO® Microshunt consists of styrene-*block*-isobutylene-*block*-styrene (SIBS), is implanted ab-externo, and has a length of 8.5 mm and a lumen of 70 μm [10–12]. It has been shown to effectively reduce IOP while it has a lower reintervention rate and bleb-related complications [13]. The risk of postoperative hypotony can be effectively reduced by introducing a monofile suture into the lumen [14]. In direct comparison with trabeculectomy, recent data for the PRESERFLO® microshunt show a weaker efficacy but a higher safety especially in patients with risk factors for hypotony and suprachoroidal bleeding [13, 15–19].

This study aims to assess changes in peripapillary RNFL thickness and BMO-MRW in a cohort of patients after PRESERFLO^®^ Microshunt implantation. Due to the extent of relative IOP lowering in early follow-up, this surgical approach is expected to cause SRDC comparable to trabeculectomy. Furthermore, we aimed to analyze the extent and duration of SRDC seen in OCT-based morphometry after PRESERFLO® microshunt implantation.

## Materials and methods

Patients, who underwent PRESERFLO® microshunt implantation at our tertiary glaucoma center between 2019 and 2022, were retrospectively reviewed to fulfill all inclusion and no exclusion criteria for this study.

Inclusion criteria were the presence of pre- and at least one postoperative SD-OCT of the ONH using the Spectralis Glaucoma Module (Heidelberg Engineering, Heidelberg, Germany). Further inclusion criteria were unambiguously diagnosed primary open angle glaucoma or secondary open angle glaucoma due to pseudoexfoliation or pigment dispersion. All included patients were scheduled for Preserflo implantation due to uncontrolled glacuoma despite exhausted tolerable topical treatment. Slit lamp examination had to show sufficient optical clarity to ensure good image quality. Required information from the patients’ medical history included at least best-corrected visual acuity (BCVA), intraocular pressure (IOP), and topicalmedication at each examination as well as at least one preoperative result of visual field testing within the last three month before surgery.

Reasons for exclusion were unsatisfactory SD-OCT image quality (Quality index < 15 dB) or acquisition and segmentation errors that could not be corrected manually. Further exclusion criteria were any kind of filtrating glaucoma surgery, vitrectomy, buckle surgery, corneal surgery including refractive surgery in the medical history. Patients with refractive errors exceeding + 3 D or below − 6 D were excluded. Amblyopia or strabism surgery were not reasons for exclusion.

Surgery was performed by experienced surgeons according to the standard operating procedures of our clinic. In all cases, a monofile suture (Dafilon 8.0) was used for temporary partial occlusion of the shunt [14]. Under subconjunctival or general anesthesia, a sub-tenon flap was opened through a limbal approach. Mitomycin-C (MMC)-soaked sponges (0.2 mg/mL) were inserted in the subconjunctival space for three minutes [20]. A triangular knife (1.0 mm) was used to perform a lamellar sclerotomy located 3 mm peripheral to the conjunctival limbus. A 25G cannula created a transscleral tunnel connecting the subsequent filtering bleb to the anterior chamber.

The microshunt was inserted into the tunnel with the fins in the scleral pocket. To achieve temporary primary partial occlusion of the shunt, a Dafilon 8.0 suture was placed intraluminally and left in place for up to two weeks postoperatively depending on IOP levels. Aqueous humour flow through the shunt was confirmed by injecting balanced salt solution into the anterior chamber and visualizing a drop of aqueous humour by a sponge at the distal end of the lumen. When correct placement of the shunt could be ensured, the conjunctiva was closed using absorbable sutures.

Follow-up examinations in the department were scheduled based on clinical need. Due to the retrospective approach of this analysis, every patient in the cohort had an individual follow-up pattern for the first postoperative year. To aggregate available data, individual follow-up examinations were grouped in the following intervals: three-month follow-up (1-135 days), 6-month follow-up (136–270 days), 12-month follow-up (271–450 days), and 18-month follow-up (451–980 days). This approach was consistent with previous publications of our group (Gietzelt et al.) [7, 21].

All patients included in this analysis had a least one preoperative SD-OCT examination of the ONH performed within one month before PRESERFLO^®^ implantation (7.2 ± 6.4 days) and at one of the follow-up examinations (3 months, 6 months, 12 months, 18 months).

Not every patient received a follow-up SD-OCT examination after each of these periods, some patients only at one of the four follow-up intervals.

Medical data, including visual acuity, corneal thickness, and topical medication was obtained from medical records. IOP values were measured by rebound tonometry (Icare Tonometer TA01i, Icare Finland Oy, Vantaa, Finland).

SD-OCT examinations were performed according to the standard operating procedures [7]. Twenty-four scans with 48 measurement points of BMO-MRW and three circular scans of RNFL thickness as well as BMO Area were calculated by the Spectralis^®^-SD-OCT platform (Heidelberg Engineering GmbH, Heidelberg, Germany), and the export tool was used for data acquisition. Both global and sectoral BMO-MRW and 3.5 mm pRNFL (T (temporal), TI (inferotemporal), TS (superotemporal), N (nasal), NI (inferotemporal, NS (superonasal)) were used for the analysis. Patients with unsatisfactory image quality and uncorrectable segmentation errors were excluded. When possible, segmentation errors were corrected manually.

### Ethical approval and statistical analysis

Based on the ruling of the Ethics Committee (EC) of the Medical Faculty and University of Cologne and according to the regulations of the professional code for Physicians, the EC waived ethical approval because of the retrospective nature of the study. Due to the retrospective nature of the study, the EC of the Medical Faculty and University of Cologne waived the need of obtaining informed consent. We adhered to all tenets of the Declaration of Helsinki.

Descriptive statistics was used for characterization of the study group. Normal distribution was tested with the D’Agostino-Pearson normality test. If RNFL and BMO-MRW values were not normally distributed, the Wilcoxon test was performed to assess significance. In the case of normal distribution, the paired t-test was performed. Spearman rho (ρ) was used to characterize correlation between change in BMO-MRW, RNFL thickness and IOP levels. Values were expressed as mean ± standard deviation of the mean (SD). Statistical significance was set at *p* < 0.05. Other high significance levels were set at *p* < 0.01 and *p* < 0.001. All analyses and data presentations were performed using Excel (Microsoft Office Excel 2016, California, USA), SPSS v. 22 (IBM Chicago, Illinois, USA), and GraphPad software (GraphPad Prism 7, Inc, La Jolla, USA).

## Results

In the period between 2019 and 2022, 142 patients underwent implantation of a PRESERFLO® microshunt at the department. Of these, three had to be excluded due to type of glaucoma and four had to be excluded due to filtrating surgery in their medical history. Another 76 patients did not fulfill the inclusion criteria because of missing baseline or follow-up OCT examination during the first two years after surgery.

In total, 59 patients could be included in the analysis. Of these, 26 patients had a gradable OCT examination after three months of follow-up (84.6 ± 24.6 days), 18 patients had available follow-up data 6 months after surgery (206.6 ± 43.3 days), 30 patients after 12 months (344.7 ± 53.7 days) and 25 patients after 18 months (652.8 ± 159.1 days). The epidemiological data of our cohort can be found in Table 1.

**Table 1:** Global morphometric parameters in study eyes

		global	p-value
BMO-MRW in µm	BL 3 months	171.15 ± 66.8	
	FU 3 months	180.78 ± 70.39	< 0.05
	BL 6 months	149.38 ± 55.44	
	FU 6 months	146.51 ± 57.59	n.s.
	BL 12 months	164.91 ± 53.84	
	FU 12 months	165.94 ± 58.24	n.s.
	BL 18 months	169.83 ± 52.69	
	FU 18 months	164.98 ± 55.85	n.s.
RNFL Thickness in µm	BL 3 months	64.33 ± 16.98	
	FU 3 months	63.19 ± 17.93	n.s.
	BL6 months	60.17 ± 14.32	
	FU 6 months	57.44 ± 15.03	< 0.05
	BL 12 months	62.7 ± 15.0	
	FU 12 months	58.8 ± 14.53	< 0.05
	BL1 8 months	62.68 ± 13.66	
	FU 18 months	58.12 ± 12.49	< 0.0001

Abbreviations: BL: baseline, FU: follow-up; BMO-MRW: Bruch Membran opening-minimum rim width; RNFL: Retinal nerve fibre layer; n.s. not significant

BMO-Area did not differ significantly (n.s.) between baseline (1.937 ± 0.428 mm²) and follow-up examinations at three (2.039 ± 0.369 mm²), six (2.031 ± 0.474 mm²), twelve (1.947 ± 0.419 mm²), and eighteen months (1.875 ± 0.423 mm²), respectively (*p* > 0.05).

Before surgery, the mean baseline IOP was 25.3 ± 6.9 mmHg. Postoperatively, mean IOP decreased to 13.7 ± 5.2mmHg (Relative Change(RC): -45.8 ± 20.6%) after three months and to 14.72 ± 4.7 (RC: -41.8 ± 18.6%) mmHg after 6 months. In later follow-up, mean IOP was 13.7 ± 4.6mmHg (RC: -45.8 ± 18.2%) at 12 months and 13.1 ± 4.5mmHg (RC: -48.2 ± 17.8%) at 18 months after PRESERFLO® implantation.

### Longitudinal follow-up of morphometric parameters and correlation to IOP

**Fig. 1:**
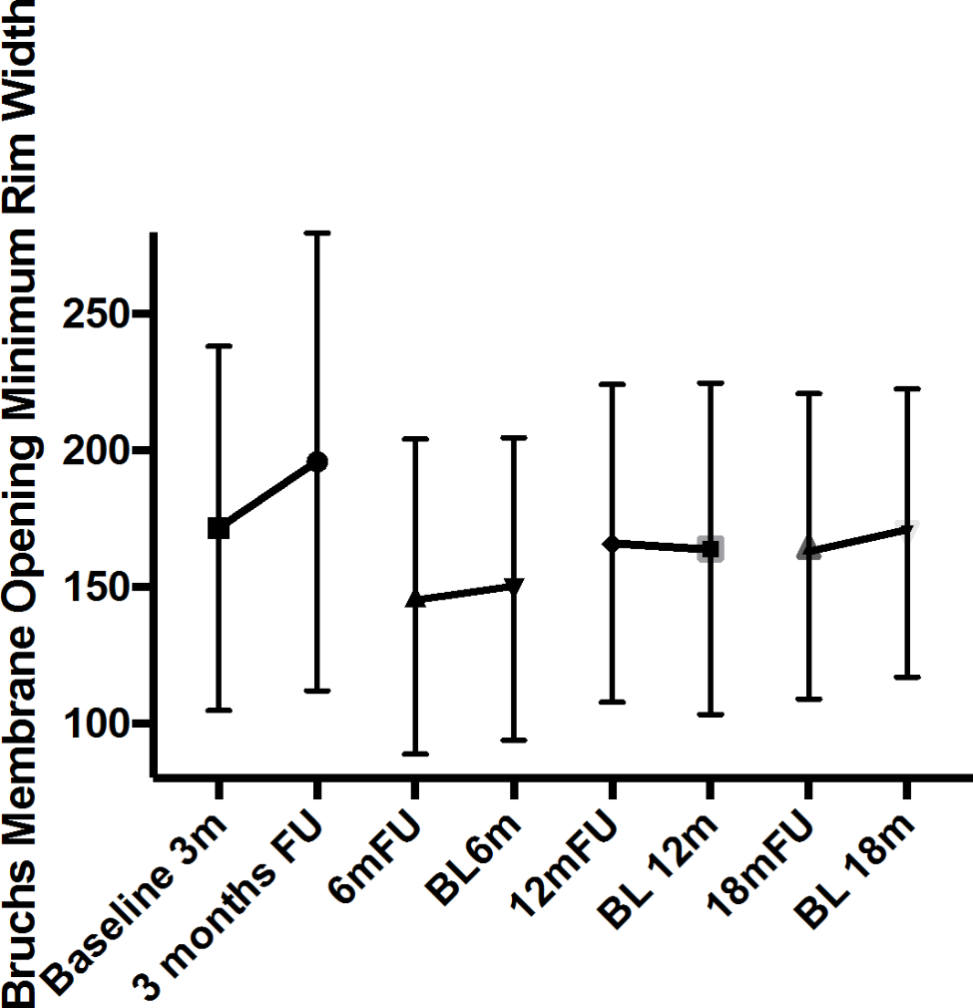
Global BMO-MRW with baseline and respective follow-up values

The parameter BMO-MRW increased significantly from a baseline thickness of 171.51 ± 66.80 μm to 180.78 ± 70.39 μm three months after surgery (*p* = 0.034) (Fig. 1). The RC was 5.4%. In later follow-up, no significant differences were found between baseline BMO-MRW and follow-up values after 6 months (baseline (BL)): 149.38 ± 55.44 follow-up (FU): 146.51 ± 57.59 RC: -2,01%; n.s.), 12 months (BL: 164.91 ± 53.84 FU: 165.94 ± 58.24 RC: +0,62%, n.s.) and 18 months (BL: 169.83 ± 52.69 FU: 164.98 ± 55.85% RC: -2.86%, n.s.).

**Fig. 2:**
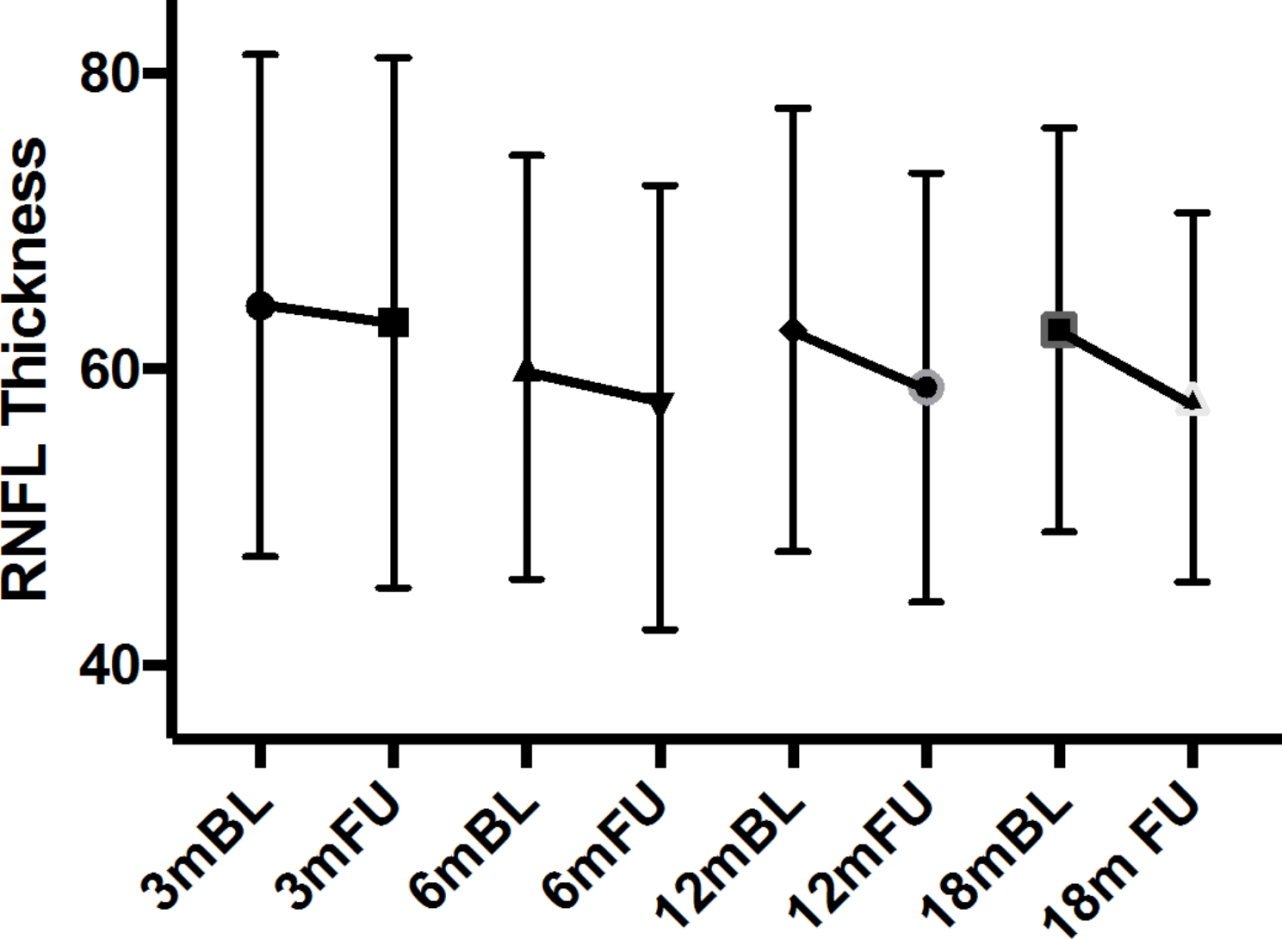
Global RNFL Thickness with baseline and respective follow-up values

Peripapillary RNFL thickness remained unchanged after three months of follow-up compared to baseline (Table 2). In later follow-ups, examinations showed a significant loss of RNFL thickness after 6 months (-2.72 ± 5.05 μm; *p* = 0.035), 12 months (-3.90 ± 8.18 μm; *p* = 0.014), and 18 months (-4.56 ± 5.01 μm; *p* < 0.001) (Fig. 2).

**Table 2:** Epidemiologic and baseline data

	Study group
	n = 59
Age (years)	75.89 ± 7.77
Caucasian	59
Actual preoperative IOP (mmHg)	24.97 ± 7.22 mmHg
Medication score initial	1.79 ± 1.85
Glaucoma subtype	
Primary open angle (n)	43
Pseudoexfoliation (n)	16
Mean Deviation (MD) in 30/2 SAP	11.72 ± 6.71
Pattern Standard Deviation (PSD)	5.89 ± 1.89
Phakic status	
Pseudophakic (n)	47
Phakic (n)	12
Spherical equivalent	-0,58 ± 1,48
Baseline BCVA (logMAR)	0.2 ± 0.1
Glaucoma stage (Hodapp-Parrish-Anderson)	
Mild	
Moderate	9
Severe	15
Perimetry not evaluable due to poor reliability parameters	18
	17

A subanalysis of all sectors of the ONH was performed both for RNFL thickness and BMO-MRW. A significant decrease of BMO-MRW was observed in the temporal-inferior sector after 18 months and in multiple sectors of RNFL thickness after 3, 6, 12, and 18 months (Table 3).

**Table 3:** Sectorial morphometric parameters in study eyes; BMO MRW and peripapillary RNFL thickness in µm, abbreviations: BL: baseline, FU: follow-up; BMO-MRW: Bruch Membran opening-minimum rim width; RNFLT = retinal nerve fibre layer thickness, values were expressed as mean ± standard deviation of the mean (SD). Statistical significance was set at *p* < 0.05 (*). Other high significance levels were set at *p* < 0.01 (**) and *p* < 0.001 (***)

BMO-MRW	Interval	Temporal	Superotemporal	Inferotemporal	Nasal	Superonasal	Inferonasal
	3 m BL	117.1 ± 56.75	123.67 ± 80.73	165.72 ± 86.49	170.52 ± 112.54	146.23 ± 107.67	180.73 ± 127.01
	FU	116.78 ± 70.96	135.53 ± 107.03	155.51 ± 100.66	185.60 ± 107.80	182.32 ± 109.77	200.73 ± 73
	6 m BL	114.84 ± 49.92	129.06 ± 73.16	109.23 ± 66.50	163.12 ± 98.92	128.4 ± 90.51	146.24 ± 76.83
	FU	95.8 ± 62.61	124.80 ± 80.79	117.90 ± 99.90	168.38 ± 89.67	154.48 ± 80.83	152.96 ± 79.27
	12 m BL	110.97 ± 58.85	109.37 ± 69.95	136.85 ± 82.55	185.50 ± 90.82	137.38 ± 64.23	183.57 ± 93.64
	FU	119.42 ± 58.22	116.74 ± 76.02	123.75 ± 69.93	176.38 ± 104.07	153.16 ± 81.31	199.55 ± 86.37
	18 m BL	123.54 ± 48.82	120.30 ± 76.08	**145.49 ± 73.86**	202.86 ± 75.85	168.36 ± 68.47	155.28 ± 107.02
	FU	119.99 ± 53.57	132.32 ± 78.40	127.70 ± 66.95*	186.69 ± 91.50	162.89 ± 89.63	180.70 ± 91.22
RNFLT	3 m BL	58.0 ± 18.49	76.45 ± 36.42	**85.55 ± 32.47**	**63.65 ± 23.95**	**70.55 ± 22.18**	69.3 ± 23.57
	FU	55.6 ± 19.17	77.25 ± 39.16	74.9 ± 35.29*	59.55 ± 25.48*	67.2 ± 22.23*	76.65 ± 52.68
	6 m BL	55.44 ± 16.38	67.28 ± 27.36	**68.33 ± 19.09**	57.44 ± 23.85	**62.5 ± 18.13**	60.72 ± 23.87
	FU	54.72 ± 16.16	62.61 ± 31.19	62.22 ± 16.1*	56.44 ± 26.41	55.78 ± 17.8**	59.22 ± 24.02
	12 m BL	**52.57 ± 17.52**	**73.3 ± 28.81**	**81.57 ± 24.39**	**58.43 ± 20.51**	**64.5 ± 20.25**	67.07 ± 18.42
	FU	49.63 ± 16.55**	68.6 ± 25.85*	75.5 ± 29.94**	54.53 ± 19.11*	58.27 ± 18.98***	65.03 ± 19.55
	18 m BL	**57.12 ± 16.06**	**79.68 ± 26.65**	**81.8 ± 27.53**	**52.8 ± 15.04**	**63.56 ± 15.98**	63.9 ± 23.46
	FU	53.2 ± 14.58***	74.04 ± 23.19***	73.12 ± 26.33***	49.36 ± 14.21**	58.12 ± 14.78***	62.0 ± 19.63

**Fig. 3:**
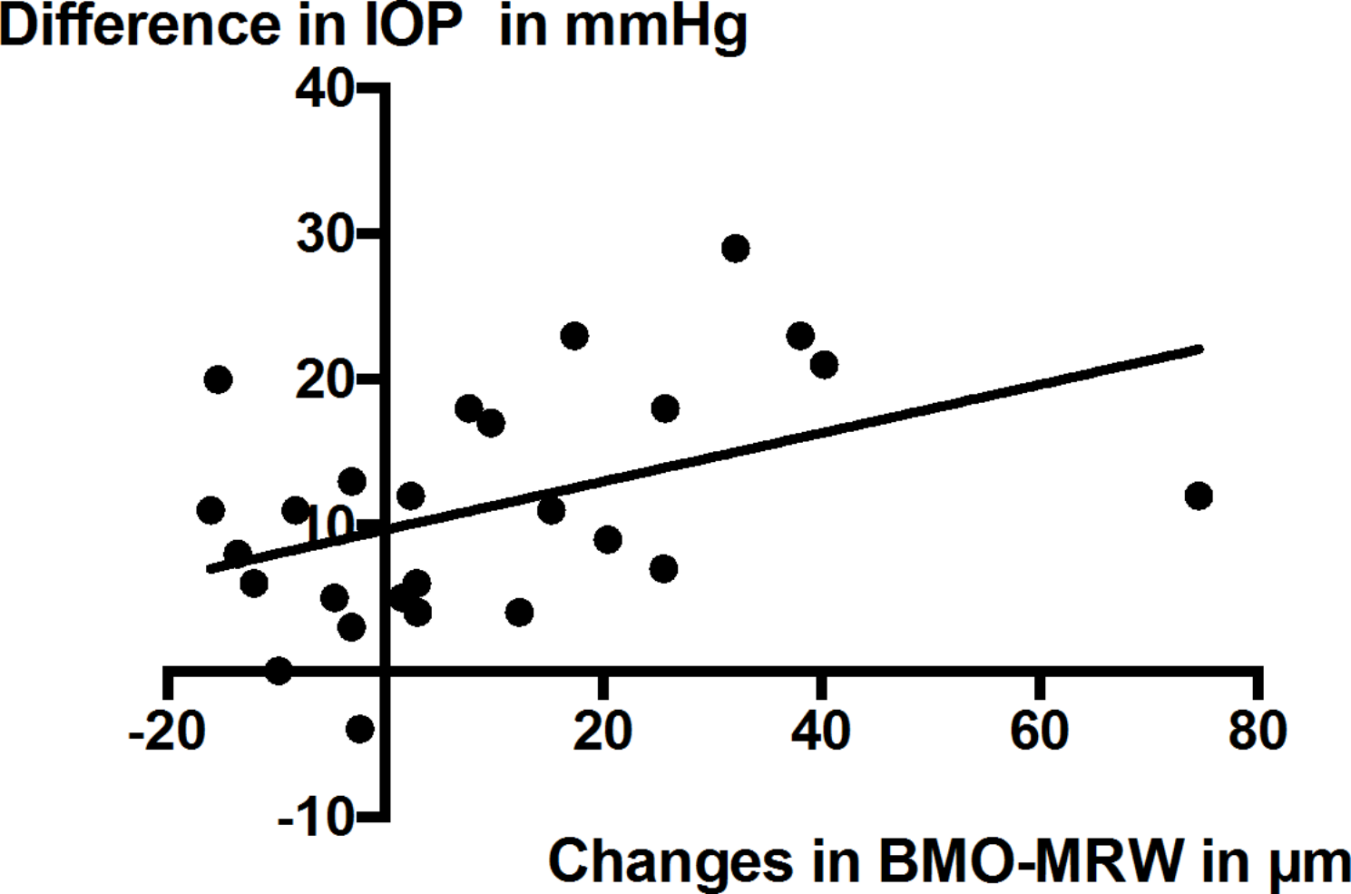
Correlation between Change in IOP and change in BMO-MRW 3 months after surgery (*r* = 0.453, *p* < 0.05)

The increase in global BMO-MRW after three months correlated significantly to the measured IOP reduction after this period (*r* = 0.453, *p* < 0.05, Fig. 3). No significant correlation was found between the difference in BMO-MRW and the absolute IOP values three months after the intervention. No correlations were found between IOP change and the difference between baseline BMO-MRW and follow-up BMO-MRW at 6, 12, and 18 months postoperatively.

A trend towards significance was observed in the correlation between the absolute IOP values 18 months after PRESERFLO® implantation and the RNFL reduction during this period (*r* = 0.38, *p* = 0.081).

Aggregation of follow-up intervals to short-term (3 and 6 months) and mid-term (12,18 months) to provide groups with a higher number of follow-up examinations did not lead to any significant changes in comparison with baseline examinations.

In addition, we compared two groups with regard to the severity of glaucoma damage based on the mean deviation of baseline perimetry examination (mild/moderate < 12 dB, *n* = 24; severe > 12 dB, *n* = 18). The increase in BMO-MRW after three months showed a tendency for higher RC in advanced glaucoma (+ 18.4 ± 23.3 μm; RC: +13.2 ± 18.8%) compared to mild/moderate glaucoma (+ 15.0 ± 22.7 μm; RC: +7.2 ± 11.6%) without reaching the threshold for significance due to the small sample size of the subgroups.

During the first 14 days after PRESERFLO® Implantation there were three cases (5.08%) of postoperative hypotony (< 5mmHg). Despite the measured IOP, no clinically relevant signs of hypotony (flat anterior chamber, choroidal detachment, choroidal folds) were observed.

## Discussion

Morphometry of the optic nerve neuroretinal rim tissue is an essential element in the evaluation of glaucoma progression. SD-OCT of the ONH can be considered a widely used clinical standard today. After glaucoma surgery, in particular after filtration surgery, the evaluation of the morphometry of the ONH in SD-OCT can be a challenge. The effect of the structural reversal of disc cupping corresponding to an increase of the neuroretinal rim tissue thickness after surgery, has already been demonstrated for traditional filtration surgery, such as trabeculectomy. Several studies measured SRDC both for CSLT and SD-OCT imaging. Also, this effect has been shown for glaucoma drainage device surgery [22]. Up to now, to our knowledge, no studies exist, which quantify this effect for so-called minimally invasive bleb surgery (MIBS).

In this study we could show a significant increase in BMO-MRW in a short-term FU period of three months for MIBS surgery with the PRESERFLO® microshunt. When analyzing longer follow-up periods of 6–18 months, no significant changes in BMO-MRW were observed. The peripapillary retinal nerve fiber layer thickness remained unchanged in early follow-up. This is consistent with observations after trabeculectomy [7].

Morphometry of the ONH is known to be influenced by IOP changes in both ways. SD-OCT imaging allows quantification of the effect [23, 24]. The substantial decrease in IOP after filtration surgery is currently considered to be the main cause of SRDC [25]. Some studies suggest strong IOP fluctuations have less or no effect on pRNFL thickness [23]. This parameter’s higher independence from IOP fluctuations allows more stable and more unbiased morphometric glaucoma progression analysis compared to BMO-based parameters [3].

The correlation between IOP and BMO-MRW is especially known for cases with strong IOP changes after filtration surgery [21, 22, 25]. Normal diurnal surgery-independent IOP fluctuations however, do not seem to have any effect on the SD-OCT parameters BMO-MRW and peripapillary RNFL thickness [4]. It is not yet sufficiently known at which IOP decrease a measurable SRDC is detectable.

ICare rebound tonometry was used according to the departments SOP in order to increase patients comfort and to reduce the risk of postoperative infection. A meta-analysis of 147 publications, including 672 eyes, revealed a meta-difference (Icare^®^ PRO-Goldmann applanation tonometry) of − 0.14 mmHg, indicating a non-significant, clinically irrelevant mean deviation (*p* = 0.335) [26].

With regard to the duration of SRDC, the results of this study differ from previous observations after different types of filtrating surgery. Also, BMO-MRW’s relative increase of 5.4% in three months follow-ups was lower than the reported change of this parameter after trabeculectomy (11.6% ± 22.6%) [7]. In summary, this study describes a less persistent and less prominent pattern of SRDC after PRESERFLO® implantation. Several hypotheses could explain these findings: Early partial occlusion of the shunt aims to avoid early postoperative hypotony and increases IOP levels in early follow-up [14]. The absence of very low IOP levels early postoperatively could reduce the development of SRDC. Another explanation could be higher IOP levels after PRESERFLO® implantation at three months follow-up compared to trabeculectomy. To test this hypothesis, a direct, preferable prospective study comparing trabeculectomy and MIBS would be desirable. Postoperative hypotony occurred in only 5.1% of patients. The rate of early postoperative hypotony seems to be higher after trabeculectomy than after PRESERFLO® implantation with temporary intraluminal occlusion [14].

The dependency between BMO-MRW and BMO-area is well known and has been described in numerous publications and as by our group. This relationship exists because, with an increasing BMO area, the retinal nerve fibers are distributed over a larger horizontal vector area, resulting in a thinner circumferential measured rim width. BMO-MRW, in turn, as shown in this study, correlates with intraocular pressure (IOP) reduction in the context of SRDC. However, BMO area does not correlate with IOP reduction. This phenomenon can be explained by the fact that BMO-MRW includes not only a horizontal vector but also a vertical vector, which is the only component influenced by SRDC.

In this study, the absolute increase in BMO-MRW after three months was similar across subgroups with varying severity of glaucoma. Thus, the extent of SRDC may not depend on the baseline BMO-MRW thickness.

In mid-term follow-up of more than 6 months after PRESERFLO® implantation, this study did not find any significant BMO-MRW increase. One explanation could be a weaker IOP-lowering effect of MIBS surgery compared to trabeculectomy: in a prospective randomized multicenter study, the PRESERFLO® microshunt performed significantly weaker in IOP lowering after one year compared to trabeculectomy with mitomycin C (14.3 mmHg vs. 11.1 mmHg) [18]. In this study, the rate of success defined as “IOP below 14 mmHg” without drop-outs was 35% for the PRESERFLO® group and 56% for the trabeculectomy group.

Another prospective study of a total of 300 patients confirmed these findings with slightly higher IOP values after 1 year of follow-up for the PRESERFLO® stent. The mean IOP was 12.9 mmHg after PRESERFLO® implantation and 11.4 mmHg after trabeculectomy [16]. Our results can be classified similarly with an average IOP of 13.7 ± 4.6mmHg at 12 months and 13.1 ± 4.5mmHg at 18 months after PRESERFLO® implantation. In our study, we found an early significant reduction of peripapillary RNFL thickness 6 months after PRESERFLO® implantation. After trabeculectomy, our previous study reported a significant RNFL thickness reduction only after 18 months postoperatively [7]. Interestingly, the amount of RNFL loss (PRESERFLO® -4.6 μm vs. trabeculectomy − 4.2 μm) was comparable at 18 months after surgery for both surgical approaches.

A stronger SRDC after trabeculectomy affecting also peripapillary RNFL thickness during the early postoperative period and potentially masking the retinal nerve fibre loss could be the reason to explain this difference between the effect after trabeculectomy and PRESERFLO® implantation:

Existing literature on SRDC has demonstrated neither a significant increase or decrease in RNFL in the early postoperative period after trabeculectomy (12 months postoperative). In contrast, this study identified a significant decrease already after the 6 months interval after shunt implantation. We therefore hypothesize that an early RNFL loss as measured for PRESERFLO may be masked by SRDC following trabeculectomy. Due to the reduction of the SRDC effect with time, the same RNFL reduction is measured after 18 months.

The tendency for a significant correlation between absolute IOP and RNFL reduction 18 months after PRESERFLO® implantation indicates that the level of long-term postoperative IOP may be crucial for the prognosis of postoperative glaucoma progression. Correspondingly, Koenig et al. reported a significant correlation between the postoperative IOP one year after filtrating surgery and the rate of progression of visual field defects [27].

Glaucoma progression as loss of neuroretinal tissue can occur focally. Therefore, the sectoral data of the OCT imaging data is crucial for structural progression analysis. Our work showed a reduction in RNFL thickness in various sectors after 3, 6, 12, and 18 months. A significant reduction in BMO-MRW was detected only in the temporal-inferior sector after 18 months. As SRDC biases BMO-MRW sectoral thicknesses for up to 18 months postoperatively, peripapillary RNFL thickness seems more suitable for an accurate assessment of glaucoma progression during this period after PRESERFLO® implantation.

The limitations of our study were the retrospective study design. It is important to consider that patients who did not have complicating events may have been less likely to come to tertiary care centers for follow-up examination. Patients with adverse events like hypotony could therefore be overrepresented in the early postoperative phase and amplify the effect. Furthermore, within the context of the retrospective study design, it was necessary to define certain follow-up groups. Nevertheless, this study may provide important additional information that is clinically relevant for the evaluation of glaucoma progression after PRESERFLO® implantation.

Furthermore, the study is limited by the lack of a control group. Previous studies have analyzed the fellow eyes’ course of BMO-based morphometry (BMO-MRW, BMO-MRA, RNFL-Thickness) following filtering surgery (glaucoma drainage device, trabeculectomy) and found no significant changes at any interval (1 week, 3, 6, 12, 18 months).

Another limitation of this study may be the exclusion of the 81 patients, which could lead to selection bias. In most cases, follow-up data were missing. This study aimed to assess real-world data to provide a basis for further clinical decisions.

Considering our results, PRESERFLO® implantation with primary temporary occlusion causes a significant transient SRDC. The phenomenon seems to be attenuated and of shorter duration when compared to trabeculectomy. The extent of SRDC may be associated with subclinical early hypotony after surgery.

## Data Availability

The datasets used and analysed during the current study available from the corresponding author on reasonable request.
